# Correlation of PD-L1 Expression with Clinicopathological and Genomic Features in Chinese Non-Small-Cell Lung Cancer

**DOI:** 10.1155/2022/1763778

**Published:** 2022-04-11

**Authors:** Yue Li, Chong Li, Ya Jiang, Xue Han, Sisi Liu, Xiuxiu Xu, Wanxiangfu Tang, Qiuxiang Ou, Hua Bao, Xue Wu, Yang Shao, Minyan Xing, Yixiang Zhang, Yuezhen Wang

**Affiliations:** ^1^Department of Medical Oncology, Harbin Medical University Cancer Hospital, 150081 Harbin, Heilongjiang, China; ^2^Department of Pulmonary and Critical Care Medicine, The Third Affiliated Hospital of Soochow University, Changzhou, Jiangsu, China; ^3^Department of Research and Development, Nanjing Geneseeq Technology Inc., Nanjing, Jiangsu, China; ^4^Haining People's Hospital, Haining, Zhejiang, China; ^5^Department of Thoracic Surgery, The First Affiliated Hospital of Dalian Medicine University, Dalian, Liaoning, China; ^6^Cancer Hospital of the University of Chinese Academy of Sciences (Zhejiang Cancer Hospital), Hangzhou, Zhejiang, China; ^7^Institute of Cancer and Basic Medicine (IBMC), Chinese Academy of Sciences, Hangzhou, Zhejiang, China

## Abstract

Programmed cell death 1 ligand 1 (PD-L1) has been approved as predictive biomarker for non-small-cell lung cancer (NSCLC) patients treated with PD-(L)1 blockade therapy. The clinical/genomic features associated with PD-L1 are not well studied. Genomic profiling of tumor biopsies from 883 Chinese NSCLC patients was performed by targeted next-generation sequencing. Immunohistochemical analysis was conducted to evaluate PD-L1 expression levels using antibodies Dako 22C3 and 28-8, respectively. Our study showed distinct correlation between PD-L1 expression and clinical/genomic characteristics when using different PD-L1 antibodies and in different histological subtypes including adenocarcinoma (ADC) and squamous cell carcinoma (SCC), respectively. PD-L1 high expression (22C3) was associated with male and lymph node metastasis only in ADC patients. Furthermore, mutations of *TP53* and *KRAS*, *KIF5B-RET* fusion, copy number gains of *PD-L1* and *PD-L2*, and arm-level amplifications of chr.12p were significantly associated with PD-L1 positive status in ADC patients. For SCC patients, the gain of *EGFR* and *MDM2* and loss of *PTPRD* were negatively associated with PD-L1 expression. We also compared our results with other studies and found conflicting results presumably because of the multiplicity of antibody clones and platforms, the difference of cutoffs for assigning PD-L1 expression levels, and the variation in study populations. Our study can help to understand the utility and validity of PD-L1 as biomarker of response to immune checkpoint inhibitors.

## 1. Introduction

Non-small-cell lung cancer (NSCLC) is the leading cause of cancer-related mortality worldwide, among which adenocarcinoma (ADC) and squamous cell carcinoma (SCC) are the most common histological subtypes [1]. Checkpoint blockade immunotherapy has had remarkable development in the last decade, and immune checkpoint inhibitor- (ICI-) targeted programmed cell death 1 (PD-1) and its ligand (PD-L1) have provided promising survival benefit for NSCLC patients [2].

To date, only PD-L1 expression, microsatellite instability (MSI), and tumor mutational burden (TMB) have been approved by the Food and Drug Administration (FDA) as predictive biomarkers for anti-PD-(L)1 therapies in patients with advanced NSCLC. However, it has been observed that MSI-high (MSI-H) or mismatch repair deficiency (dMMR) rarely appears in lung adenocarcinoma [3], and the predictive capacity of PD-L1 or TMB testing alone is insufficient [4–6]. Several effectors compromise the predictive accuracy of PD-L1 expression, including the heterogeneity of PD-L1 expression within tumor site, the multiplicity of antibody clones and platforms, and the difference of cutoffs for assigning PD-L1 expression levels [7]. Numerous studies have proved that the predictive value of PD-L1 expression is also affected by clinical, pathological, and molecular characteristics, such as age, sex, tumor site, and genomic alterations [8, 9]. It has been reported that genomic alterations of *KRAS*, *TP53*, *BRAF*, and *MET* are associated with increased expression of PD-L1in NSCLC patients [10, 11], while *EGFR* and *STK11* mutations are correlated with negative/low PD-L1 expression [11, 12], whereas genomic features related to PD-L1 are different among various study populations, antibody clones, and experimental platforms.

Since genetic testing is widely applied in genomic profiling to identify druggable alterations of NSCLC, we aimed to find the correlation between genomic alterations, TMB and PD-L1 expression, and whether genomic alterations can be used in discriminating PD-L1 level. Herein, we performed a retrospective study correlating the presence of clinical characteristics, genomic alterations, and PD-L1 expression in Chinese NSCLC patients including ADC and SCC, providing data of the association between PD-L1 expression and genomic features when using different PD-L1 antibodies and in different histological subtypes for identifying potential anti-PD-L1 treatment-related biomarkers in NSCLC.

## 2. Materials and Methods

### 2.1. Patients and Samples

We retrospectively reviewed Chinese NSCLC patients who underwent next-generation sequencing (NGS) with a cancer-relevant gene panel of 139 or 425 genes (Supplementary Table S1) and PD-L1 testing on the same tissue sample at the Harbin Medical University Cancer Hospital, The Third Affiliated Hospital of Soochow University, The First Affiliated Hospital of Dalian Medicine University, and Cancer Hospital of the University of Chinese Academy of Sciences between November 2018 and July 2019. All NSCLC patients were pathologically confirmed. Exclusion criteria were poor-quality tumor samples and incomplete clinicopathological features. The specimen slides less than 100 viable tumor cells or the percentage of tumor cells <10% were also excluded. The study was approved by the ethical committees of all participating hospitals. All patients have provided written informed consent. Overall, 883 specimens were collected and analyzed for clinicopathological characteristics including age, sex, histology type, pathological stage, and tumor site. All the samples were shipped to the central laboratory of a clinical testing center (Nanjing Geneseeq Technology Inc., China) for genetic and PD-L1 testing.

### 2.2. PD-L1 Testing

PD-L1 immunohistochemistry (IHC) staining was performed using monoclonal mouse anti-human PD-L1 antibody (clone 22C3, *n* = 750, Dako: Cat No. M3653) or monoclonal rabbit anti-PD-L1 antibody (clone 28-8, *n* = 133, Abcam: Cat No. ab205921). A minimum of 100 viable tumor cells must be present in the specimen slide for the PD-L1 expression to be calculated with complete or partial membrane staining. For samples stained by clone 22C3, their PD-L1 expression levels were defined by the tumor proportion scores (TPS) to be negative (<1%), low expression (1-49%), and high expression (≥50%), respectively, which was consistent with the cutoffs used in pembrolizumab clinical trials [13, 14]. Similarly, the PD-L1 expression levels for samples stained by clone 28-8 were defined as negative (<1%), low expression (1-9%), and high expression (≥10%), according to the nivolumab clinical trials [15]. The representative images of immunohistochemical staining for PD-1 with clone 22C3 or clone 28-8 from ADC or SCC patients were shown in Supplementary Figure S1.

### 2.3. Next-Generation Sequencing and Bioinformatic Analysis

Genomic DNA was extracted from tumor tissues, followed by sequencing library preparation according to published protocols [16]. Hybridization capture-based targeted NGS, which was used to selectively target 139 or 425 cancer-specific genes (clone 22C3: *n*_139_ = 122 and *n*_425_ = 628; clone 28-8: *n*_139_ = 7 and *n*_425_ = 126), was performed on the Illumina Hiseq platform (Illumina, San Diego, CA). The samples were analyzed as previously described to identify genomic alterations and were presented only when the percentage of tumor cells ≥10% [17]. Raw sequencing data were first demultiplexed by bck2fastq and then trimmed by trimmomatic as part of the quality control (QC) protocol [18]. The qualified reads were then mapped onto the human reference genome (GRCh37/UCSC hg19) using the Burrows-Wheeler Aligner (bwa-mem) [19]. PCR duplicates were removed using Picard suite (http://picard.sourceforge.net); base quality score recalibration and local realignment were performed using the Genome Analysis Toolkit (GATK, version 3.4) [20]. MuTect [21] and SCALPLE (http://scalpel.sourceforge.net) were then applied to identify mutations and structure variations (SVs). For oncogenic alterations calling, we used oncology knowledge base (OncoKB), a database for the oncogenic effects and treatment implications of cancer-related alterations, to identify candidate mutations and SVs in data from both the 139 and the 425 panels.

Copy number variations (CNVs) were only analyzed with data from patients subjected to the 425-panel sequencing, as the small number of cancer genes in the 139 panel provided challenges in accurately identifying CNVs. Gene-level and arm-level copy numbers were calculated with a reported pipeline [22, 23]. We used a noise reduction model built from a pool of normal samples to process the read count from targeted regions of interest. A fold change threshold of 2 and 0.5 was used to identify gene CNV gain and loss, respectively. The arm-level CNV was identified if more than 50% of the corresponding chromosome segment length was either deleted or amplified.

### 2.4. Statistical Analysis

Statistical analyses for age were performed using the Wilcox test in group 1 (TPS = 1% cutoff for clone 22C3), group 2 (TPS = 50% cutoff for clone 22C3), group 4 (TPS = 1% cutoff for clone 28-8), and group 5 (TPS = 10% cutoff for clone 28-8) and Jonckheere trend test in group 3 (TPS of <1%, 1-49%, and ≥50% for clone 22C3) and group 6 (TPS of <1%, 1-9%, and ≥10% for clone 28-8), respectively. We have defined the groups because PD-L1 IHC 22C3 is indicated as an aid in identifying NSCLC patients for different anti-PD-1/PD-L1 drugs, such as pembrolizumab (TPS ≥ 1%) or cemiplimab (TPS ≥ 50%) [24, 25]. And PD-L1 IHC 28-8 is indicated as an aid in identifying NSCLC patients for treatment with nivolumab and atezolizumab (TPS ≥ 1%) [26]. Between-group differences of sex, metastatic sites and genomic alterations were examined using Fisher's exact test in groups 1, 2, 4 and 5 and the Cochran-Armitage test for trend test in group 3 and 6. As a minimal requirement, every mutation must be presented in at least six patients for each group. Correlations between TMB and PD-L1 (clone 22C3) expression were examined by using Spearman's rank correlation. The two-sided *p* values < 0.05 were considered statistically significant, and adjust *p* (adj. *p*) values with correcting for the false discovery rate (FDR) < 0.25 were highlighted. Statistical analyses were performed with SPSS version 25.0 (IBM) software and R version 3.3.3 software.

## 3. Results

### 3.1. Samples and Clinical Characteristics

The clinicopathological characteristics of all 883 NSCLC patients were analyzed (Table 1). Among the 750 patients with PD-L1 expression stained by clone 22C3 (median age = 61), 373 (49.7%) patients were PD-L1 positive (TPS ≥ 1%). For the remaining 133 patients (median age = 60) with PD-L1 expression stained by clone 28-8, 79 patients were PD-L1 positive (TPS ≥ 1%).

**Figure 1:**
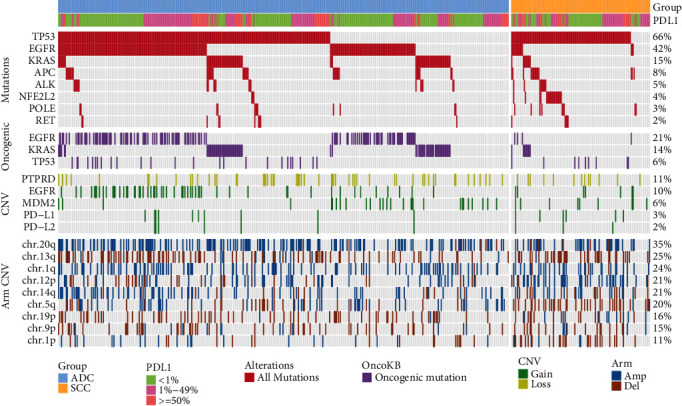
PD-L1 expression (22C3 determined) correlated with clinicopathological characteristics. (a and b) Male and metastasis significantly correlated with PD-L1 expression in ADC (*p* = 0.006 and 0.026). (C) Lymph node metastasis significantly correlated with PD-L1 expression in SCC (*p* = 0.031). TPS: tumor proportion score.

The frequency of PD-L1 positive was 47.8% (264/552, clone 22C3) and 43.4% (56/99, clone 28-8) in ADC and 52.4% (75/143, clone 22C3) and 57.1% (8/14, clone 28-8) in SCC (Table 1). PD-L1 high expression (clone 22C3, TPS ≥ 50%) for ADC patients was more common in male (*p* = 0.006 in group 2) which was confirmed by binary logistic regression analysis with age and sex (*p* = 0.009, data not shown) and metastatic samples (*p* = 0.026 in group 2) (Figures 1(a) and 1(b)). In addition, there was no statistically significant relationship between PD-L1 expression and tumor stage (data not shown). In the SCC patients, lymph node metastasis was enriched in PD-L1-positive group (clone 22C3, TPS ≥ 1%) compared to samples with other metastatic sites (*p* = 0.031 in group 1) (Figure 1(c)). In clone 28-8-detected PD-L1 level, no significant association between PD-L1 expression and the clinicopathological characteristics was identified for ADC patients. There were only 14 SCC patients who had PD-L1 testing by clone 28-8, so we could not analyze their features with PD-L1 expression.

### 3.2. Association between Oncogenic Genomic Alterations and PD-L1 Expression

**Figure 2:**
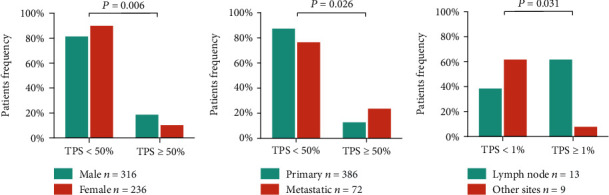
Coalteration plot for genomic alteration significantly correlated with PD-L1 expression (TPS of <1%,1-49%, ≥50%, 22C3 determined) in ADCs (*n* = 552) and SCC (*n* = 119). Significant gene mutation, oncogenic mutation, CNV, and arm CNV were showed.

Further study of the association between genomic alterations and PD-L1 expression (clone 22C3) in ADC showed that the mutations of *TP53*, *EGFR*, *KRAS*, *RET*, and *POLE* were the most relevant to PD-L1 expression (Figure 2). The Cochran-Armitage trend test showed that PD-L1 expression (clone 22C3) was positively associated with *TP53* and *KRAS* and negatively associated with *EGFR*, *RET*, and *POLE*.

As oncogenic mutations may have more direct functions in tumorigenesis, we then used a gene list from OncoKB to search for the oncogenic alterations in our data and observed that oncogenic mutations of *EGFR*, *KRAS*, and *TP53* were significantly related with PD-L1 expression (clone 22C3) in ADC (Figure 2 middle). We then focused on the difference between oncogenic mutations of *EGFR* L858R and exon 19 in-frame deletion (19Del) in association with PD-L1 expression, as they are the two most well-studied oncogenic mutations showing distinct functions in mediating carcinogenesis [27, 28]. Interestingly, the frequency of cases with TPS < 1% was higher in *EGFR*^19Del^ than *EGFR*^L858R^ (63% vs. 51%). Meanwhile, PD-L1 positive (TPS ≥ 1%) was enriched in patients with *EGFR*^L858R^ but significantly inversely associated with *EGFR*^19Del^ (*p* = 0.021 and adj.*p* = 0.047 in group 2, Supplementary Table S2). Furthermore, our results suggested that the association between PD-L1 expression and other *EGFR* oncogenic mutations (*EGFR*^other^) such as exon 20 insertion and T790M was similar to *EGFR*^19Del^. Additionally, all the oncogenic mutations of *KRAS* (*p* < 0.001 and adj.*p* = 0.004 in group 3) and especially *KRAS*^G12^ which was the most common *KRAS* oncogenic mutation (*p* = 0.002 and adj.*p* = 0.007 in group 3) had higher mutation rates when PD-L1 expression (clone 22C3) was higher (Supplementary Table S2).

In ADC patients, *EML4-ALK*, *VCL-ALK*, *KIF5B-RET*, *ERC1-ROS1*, and *MYO5A-ROS1* were the most frequently occurred gene fusions. Among all these fusion genes, *KIF5B-RET* was the only significant fusion gene that was positively associated with PD-L1 expression (clone 22C3, *p* = 0.003 and adj.*p* = 0.228 in group 3, Supplementary Table S2).

Interestingly, *POLE* mutation was enriched in the PD-L1-positive/ PD-L1-high (clone 22C3) group in SCC, but not in the PD-L1-negative group in ADC (Figure 2). As an enzyme involved in DNA repair, *POLE* mutations have been associated with disruption of the exonuclease activity required for proofreading function, which results in a high TMB level and is vulnerable to ICIs [29, 30]. In our data, the different correlation of *POLE* with PD-L1 expression in SCC and ADC indicated distinct responses of SCC and ADC patients with *POLE* mutations upon ICI treatment. Further studies should be conducted to validate this hypothesis. Additionally, oncogenic *TP53* mutations exhibited lower frequency in the SCC population with positive PD-L1 expression (clone 22C3, *p* = 0.049, Supplementary Table S2) but not in the ADC population. In SCC, PD-L1 expression (clone 22C3) was positively associated with *ALK* and *NFE2L2*, and negatively associated with *APC* (Figure 2).

As TMB is used as an independent biomarker for immunotherapy, we further analyzed the relation between TMB and PD-L1 expression and found that TMB did not correlate with PD-L1 expression (Supplementary Figure S2).

### 3.3. Correlation between CNVs and PD-L1 Expression

Then, the association between CNVs and PD-L1 expression (clone 22C3) was studied (Figure 2). Within the aforementioned 464 ADC patients, gene copy gains in *PD-L1* and *PD-L2* were significantly associated with PD-L1-positive status (*p* < 0.001 and adj.*p* = 0.016 and *p* < 0.001 and adj.*p* = 0.021, respectively) (Supplementary Table S3). *PD-L2* is also a ligand of *PD-1*, which mediates T cell activity inhibition [31]. It has been reported that *PD-L2* expression was correlated with *PD-L1* in esophageal squamous cell carcinoma [32], indicating a functional relation of *PD-L1* and *PD-L2* in tumorigenesis. Therefore, we focused on *PD-L2* and found its copy number gain to be significantly in agreement with PD-L1 expression, implying an interaction of *PD-L1* and *PD-L2* signaling. Further studies are needed to better understand the correlation between *PD-L1* and *PD-L2*. Copy number gains of *MDM2* were inversely associated with PD-L1 high expression (TPS ≥ 50%) in our data. Meanwhile, the arm-level amplifications of chr.1q (*p* = 0.012) and deletions of chr.1p (*p* = 0.041), chr.5q (*p* = 0.007), and chr.12p (*p* = 0.028) were negatively correlated with PD-L1 (clone 22C3) expression in ADC, and only amplification of chr.12p (*p* = 0.044) was associated with PD-L1 positive status (Supplementary Table S4).

For the 119 SCC patients, gain of *EGFR* and *MDM2* (*p* = 0.040 and adj.*p* = 0.234 and *p* = 0.044 and adj.*p* = 0.234, respectively) and loss of *PTPRD* (*p* = 0.015 and adj.*p* = 0.234) were negatively associated with PD-L1 expression (Supplementary Table S3). It has been reported that patients with *MDM2* amplification have higher prevalence of hyperprogression when treated with anti-PD-(L)1 immunotherapy [33]. Additionally, our data showed a significantly inverse correlation between *MDM2* copy number gain and PD-L1 expression (clone 22C3) in both ADC and SCC patients, which indicated a functional regulation of PD-L1 by *MDM2* in NSCLC. PD-L1 expression (clone 22C3) was negatively associated with copy number amplification in chr.14q (*p* = 0.032) and chr.20q (*p* = 0.026) and deletion in chr.19p (*p* = 0.025) but positively associated with chr.9p amplification (*p* = 0.008 and adj.*p* = 0.211) and chr.13q deletion (*p* = 0.019) (Supplementary Table S4).

In ADC patients, the correlation between CNVs and PD-L1 expression (clone 28-8) showed that *NKX2-1* (*p* < 0.001) gain was associated with PD-L1 expression low status, and deletion of chr.9q (*p* = 0.038) was negatively correlated with PD-L1 expression. Additionally, deletions of chr.19q and 19p were significantly associated with PD-L1 low status (both *p* = 0.039 and *p* = 0.031, respectively) (Supplementary Table S5).

## 4. Discussion

We retrospectively studied PD-L1 expression and genomic alterations to identify the correlation between them in Chinese NSCLC patients. Since each ICIs has its own antibody and clinical cutoffs, which was specially developed and associated with different clinical trials, we divided the patients accordingly into two cohorts (clone 22C3 or clone 28-8).

In our study, higher PD-L1 expression (clone 22C3) in male ADC patients was identified, which was consistent with the previous studies [8, 34]. Our data also showed that PD-L1 expression (clone 22C3) was significantly higher in tissue derived from a metastatic site compared to the primary tumor in ADC patients. This finding suggests that PD-L1 expression may be higher in advanced disease than in earlier stages [35]. Additionally, the proportion of cases with PD-L1 high expression (clone 22C3) among metastatic sites is higher in lymph node than other sites in SCC patients but not in ADC patients. PD-L1 expression may vary among different tumor sites, indicating that repeat biopsy and PD-L1 staining can be conducted to improve the predictive capacity of PD-L1 [36].

We also observed genomic alterations correlating with PD-L1 expression (clone 22C3). In the ADC population, alterations of *TP53* and *KRAS* were positively associated with PD-L1 expression. In contrast, *EGFR* and *RET* alterations were associated with PD-L1 negatively. The association between PD-L1 expression (clone 22C3) and oncogenic alterations from OncoKB provides information on the prognostic and predictive significance of somatic alterations, which can be used to optimize treatment decisions [37]. For further investigation of oncogenic alterations, we also find the difference between *EGFR*^19del^ and *EGFR*^L858R^, as PD-L1 positive was enriched in patients with *EGFR*^L858R^ but inversely associated with EGFR^19Del^. Conversely, all the *KRAS* oncogenic mutations and especially *KRAS*^G12^ are correlated with high PD-L1 expression (clone 22C3). Interestingly, *TP53* oncogenic mutations have no association with PD-L1 expression in ADC patients but are associated with negative PD-L1 expression (clone 22C3) in SCC patients. Previous study has shown that *TP53* mutations affected immune checkpoints expression, T cell infiltration, and tumor immunogenicity in lung ADC [38]. Besides, a research has shown that *TP53* oncogenic mutations were enriched in PD-L1-high group of American patients with nonsquamous NSCLC which was different from our study [12]. Moreover, other studies showed a correlation between *TP53* mutations and PD-L1 expression as well as response to ICIs in ADC [39] or SCC [10]. Since *TP53* mutations increased numbers of somatic mutations and expression of neoantigens, high TMB is more likely to benefit from immunotherapy [10, 40]. The distinct correlation between PD-L1 and *TP53* mutations may be due to the difference between populations, experimental platforms, and methods of PD-L1 testing. It has been reported that amplifications in *MDM2* and *MDM4* are associated with hyperprogression with anti-PD-(L)1 therapy [38]. In our study, *MDM2* amplification is correlated with low PD-L1 expression (clone 22C3). In our data, the different correlation of *POLE* with PD-L1 expression in SCC and ADC may indicate distinct responses of SCC and ADC patients with *POLE* mutations upon ICI treatment.

There was no significant association between PD-L1 expression and the clinicopathological characteristics in ADC patients detected with PD-L1 clone 28-8. And the genetic alterations related to PD-L1 were different in clone 22C3 and clone 28-8. For PD-L1 antibody clone 28-8-detected ADC patients, *NKX2-1* gain and deletions of chr.9p were negatively correlated with PD-L1 expression. Additionally, deletions of chr.19p and 19q were significantly enriched in PD-L1-low group. Interestingly, no mutation was found to be related with 28-8-detected PD-L1 expression. These differences may be caused by the limited sample size of patients with 28-8-detected PD-L1 level (*n* = 93) and the different epitopes and cutoffs of 28-8 and 22C3 as the previous studies [41, 42].

Genomic features related to PD-L1 are different among populations and experimental platforms. We have reviewed previous studies regarding the association between molecular features and PD-L1 expression using different antibodies (Table 2). Using the SP142 antibody with the cutoff of ≥1% and ≥50% for TPS, and at ≥1% and ≥10% for immune proportion score (IPS), PD-L1 was correlated negatively with *EGFR* mutations and positively with *KRAS*, *BRAF*, and *MET* mutations and *ROS1* translocations, in Chinese NSCLC patients [11]. On the other hand, using a rabbit polyclonal anti-PD-L1 antibody and a median histological score value of 30 as the cutoff point, the presence of *EGFR* mutations was found to be associated with increased PD-L1 expression in Japanese NSCLC patients [43]. It also reported that PD-L1 expression was associated *TP53*, *KRAS*, and *STK11* mutations when using the primary antibody 5H1 with the cutoff at ≥1% in Germany NSCLC patients [10]. Using different antibodies, including E1L3N, 22C3, 28–8, SP142, and SP263, PD-L1 subgroups were defined as negative (PD − L1 < 1%), intermediate (PD-L1 1-49%), and high (PD − L1 ≥ 50%), and mutations in *KRAS*, *TP53*, and *MET* were demonstrated to be associated with PD-L1 high expression and *STK11* mutations associated with PD-L1 negativity in American patients with lung adenocarcinomas [44]. In addition, in a Chinese NSCLC cohort, it was also confirmed that the *TP53/KRAS* subgroup manifested exclusive increased expression of PD-L1 and a highest proportion of *PD-L1*^+^/*CD8A*^+^ [39]. Furthermore, PD-L1 positivity was correlated with copy gain of *CD274* (*PD-L1*) and *PDCD1LG2* (*PD-L2*) in American nonsquamous NSCLC patients in a study using clone E1L3N with the cutoff at ≥1% [12]. Moreover, 11q13 amplification was found to be associated with high PD-L1 expression in Chinese NSCLC patients in a study using the 22C3 and 28-8 antibody at TPS cutoff values of ≥1% [45]. Recent studies have reported that PD-L1 expression is associated with *STK11* mutations, *KEAP1* mutations, *APC* mutations, and *JAK2* gain [12, 46], which was not observed in our study. These conflicting results are presumably because of the multiplicity of antibody clones and platforms, the difference of cutoffs for assigning PD-L1 expression levels, and the variation in study populations. TMB and PD-L1 expression are the two independent predictive biomarkers for anti-PD-1/PD-L1 therapy [34]. In line with the previous findings, we find no association between TMB and PD-L1 expression [44, 47].

Our model, which used a combination of seven markers, including *EGFR* oncogenic mutation, *KRAS* oncogenic mutation, *PD-L2* gain, *PD-L1* gain, *MDM2* gain, chr.1q amplification, and chr.20q amplification, showed good performance to associate PD-L1 expression (TPS = 50% cutoff for clone 22C3) in ADC patients. Based on the model results, patients with PD-L1 high had a significantly longer PFS compared to low PD-L1 expression. These results indicated that the model can help to understand PD-L1 level for patients who have no tissue available for PD-L1 IHC.

There are still several limitations of our study which should be considered and further studied in the future. Firstly, not all enrolled patients provided completely clinical characteristics, especially the smoking status, the stage, and the treatment history. Previous study observed an association of PD-L1 high expression with smoking status [48, 49] which may be related to the alteration of tumor microenvironment by smoking [9], whereas no smoking data was available in our study which may be regarded as an issue of the real-world data [50, 51]. Additionally, we did not observe a statistically significant relationship between PD-L1 expression and tumor stage in the ADC which may due to the small sample size of patients with stage I-II lung cancer in our study. Further research is warranted to study the association between PD-L1 expression and patients' clinicopathological characteristics. Secondly, our model only included patients with clone 22C3-detected PD-L1 expression with the 50% cutoff, and researches on different PD-L1 antibodies and their cutoffs were still needed. Furthermore, a large population and more genomic features should be studied in order to further understand which patients may respond to ICIs.

## 5. Conclusions

This study revealed the correlation between PD-L1 expression, clinical features, genomic alterations, and TMB in Chinese NSCLC patients and highlighted the discordance of the association between PD-L1 expression and genomic features when using different PD-L1 antibodies and different histological subtypes including ADC and SCC. PD-L1 subgroups were defined different groups according to the cutoffs of approved treatment for NSCLC patients. Moreover, our study is comprehensive and expensive since our analysis uses a large database of more than 880 Chinese NSCLC cases, and we compared our results with other studies and found conflicting results. Our results help to understand the relationship between genomic alterations and PD-L1 expression and may provide a novel idea for application of molecular features.

## Figures and Tables

**Table 1:** Clinicopathological characteristics and PD-L1 expression (clone 22C3 and clone 28-8) of all patients (*n* = 883).

Characteristic	PD-L1 (clone 22C3)			PD-L1 (clone 28-8)		
	Total	Negative	Positive	Total	Negative	Positive
Overall, *n*	750	377	373	133	54	79
Age, median, years	61 (17-91)			60 (27-84)		
<60, *n* (%)	337 (45)	182	155	66 (50)	29	37
≥60, *n* (%)	413 (55)	195	218	67 (50)	25	42
Sex, *n* (%)						
Male	484 (65)	239	245	83 (62)	33	50
Female	266 (35)	138	128	50 (38)	21	29
Histology, *n* (%)						
ADC	552 (74)	288	264	99 (74)	43	56
SCC	143 (19)	68	75	14 (11)	6	8
ASC	13 (2)	4	9	2 (1)	0	2
LCC	4 (<1)	2	2	1 (<1)	0	1
Other	4 (<1)	4	0	3 (2)	2	1
Unknown	34 (5)	11	23	14 (11)	3	11
Stage, *n* (%)						
I-II	30 (4)	15	15	5 (4)	1	4
III-IV	484 (65)	246	238	75 (56)	33	42
Unknown	236 (31)	116	120	53 (40)	19	34

Abbreviations: PD-L1: programmed cell death-ligand 1; ADC: adenocarcinoma; SCC: squamous cell carcinoma; ASC: adenosquamous carcinoma; LCC: large cell carcinoma.

**Table 2:** Summary of studies on molecular association of PD-L1 expression using the different antibody.

	No.	Pts	Region	Antibody	Cutoff	PD-L1 expression	
						Negative association	Positive association
Li et al. (11)	1370	NSCLC	China	SP142	≥1% and ≥50% for TPS	*EGFR* mutations	*KRAS*, *BRAF*, and *MET* mutations and *ROS1* translocations
Azuma et al. (44)	164	NSCLC	Japan	Rabbit polyclonal antibodies	A median histological score value of 30	—	*EGFR* mutations
Scheel et al. [10]	1255	NSCLC	German	5H1	≥1% for TPS	—	*TP53*, *KRAS*, and *STK11* mutations
Schoenfeld et al. [44]	1586	ADC	America	E1L3N, 22C3, 28–8, SP142, and SP263	≥1% and ≥50% for TPS	*EGFR* and *STK11* mutations	*KRAS*, *TP53*, and *MET* mutations
Dong et al. [39]	462	ADC	China	SP142	≥50% for TPS	—	*TP53/KRAS* comutation
Lamberti et al. [12]	909	NSCLC	America	E1L3N	≥1% for TPS	*STK11*, *EGFR*, *CTNNB1*, *APC*, and and *SMARCA4* mutations; loss of *CD274*, *PDCD1LG2*, *JAK2*, and the 9p24.1 locus	*CD274* and *PDCD1LG2* copy number gain
Zhang et al. [46]	568	NSCLC	China	22C3 and 28-8	≥1% for TPS	*EGFR* mutations	*MET*, *RET*, *ROS1*, and *TP53* mutations and 11q13 amplification

-: not application; ADC: adenocarcinoma; NSCLC: non-small-cell lung cancer; PD-L1: programmed cell death ligand 1; Pts: patients; TPS: tumor proportion score.

## Data Availability

All data relevant to the study are included in the article or uploaded as supplementary information.

## Abbreviations

ADC: Adenocarcinoma
SCC: Squamous cell carcinoma
TMB: Tumor mutational burden
NSCLC: Non-small-cell lung cancer
PD-1: Programmed cell death 1
PD-L1: Programmed cell death 1 ligand 1
ICIs: Immune checkpoint inhibitors
MSI: Microsatellite instability
FDA: Food and Drug Administration
MSI-H: Microsatellite instability-high
dMMR: Mismatch repair deficiency
TPS: Tumor proportion scores
NGS: Next-generation sequencing
QC: Quality control
SVs: Structure variations
CNVs: Copy number variations
OncoKB: Oncology knowledge base
FDR: False discovery rate
ROC: Receiver operating characteristic
DCB: Durable clinical benefit
NDB: No durable benefit
HR: Hazard ratio.

## Supplementary Materials

Supplementary Materials Supplementary Figure S1: representative images of immunohistochemical staining for PD-1 with 22C3 and 28-8 from ADC or SCC patients. (A-C) PD-L1 expression (clone 22C3) in ADC: (A) TPS < 1%, (B) TPS 1-49%, and (C) TPS ≥ 50%. (D-F) PD-L1 expression (clone 22C3) in SCC: (D) TPS < 1%, (E) TPS 1-49%, and (F) TPS ≥ 50%. (G-I) PD-L1 expression (clone 28-8) in ADC: (G) TPS < 1%, (H) TPS 1-9%, and (I) TPS ≥ 10%. (J-L) PD-L1 expression (clone 28-8) in SCC: (J) TPS < 1%, (K) TPS 1-9%, and (L) TPS ≥ 10%. PD-L1: programmed cell death-ligand 1; ADC: adenocarcinoma; SCC: squamous cell carcinoma; TPS: tumor proportion score. Scale bar: 100 *μ*m. Supplementary Figure S2: scatter plot of PD-L1 expression (clone 22C3) with TMB in ADC. TMB does not correlate with PD-L1 expression (*n* = 464, Pearson's correlation = 0.060). Triangle represents specimen of each patient. Supplementary Table S1: gene list of 139 panel and 425 panel. Supplementary Table S2: genetic mutations and PD-L1 expression (clone 22C3) in ADC (*n* = 552) and SCC (*n* = 119) patient samples. Supplementary Table S3: gene copy number variations and PD-L1 expression (clone 22C3) in ADC (*n* = 464) and SCC (*n* = 119) patient samples. Supplementary Table S4: arm-level copy number variations and PD-L1 expression (clone 22C3) in ADC (*n* = 464) and SCC (*n* = 119) patient samples. Supplementary Table S5: genomic alterations and PD-L1 expression (clone 28-8) in ADC (*n* = 93) patient samples.

## References

[1] Molina J. R., Yang P., Cassivi S. D., Schild S. E., Adjei A. A. (2008). Non-small cell lung cancer: epidemiology, risk factors, treatment, and survivorship. *Mayo Clinic Proceedings*.

[2] Heigener D. F., Reck M. (2018). Advanced non-small cell lung cancer: the role of PD-L1 inhibitors. *Journal of Thoracic Disease*.

[3] Zhao P., Li L., Jiang X., Li Q. (2019). Mismatch repair deficiency/microsatellite instability-high as a predictor for anti-PD-1/PD-L1 immunotherapy efficacy. *Journal of Hematology & Oncology*.

[4] Reck M., Rodríguez-Abreu D., Robinson A. G. (2016). Pembrolizumab versus chemotherapy for PD-L1–positive non–small-cell lung cancer. *The New England Journal of Medicine*.

[5] Hirsch F. R., McElhinny A., Stanforth D. (2017). PD-L1 immunohistochemistry assays for lung cancer: results from phase 1 of the blueprint PD-L1 IHC assay comparison project. *Journal of Thoracic Oncology*.

[6] Miao D., Margolis C. A., Vokes N. I. (2018). Genomic correlates of response to immune checkpoint blockade in microsatellite-stable solid tumors. *Nature Genetics*.

[7] Haragan A., Field J. K., Davies M. P. A., Escriu C., Gruver A., Gosney J. R. (2019). Heterogeneity of PD-L1 expression in non-small cell lung cancer: implications for specimen sampling in predicting treatment response. *Lung Cancer*.

[8] Zhang M., Li G., Wang Y. (2017). PD-L1 expression in lung cancer and its correlation with driver mutations: a meta-analysis. *Scientific Reports*.

[9] Lan B., Ma C., Zhang C. (2018). Association between PD-L1 expression and driver gene status in non-small-cell lung cancer: a meta-analysis. *Oncotarget*.

[10] Scheel A. H., Ansén S., Schultheis A. M. (2016). PD-L1 expression in non-small cell lung cancer: correlations with genetic alterations. *Oncoimmunology*.

[11] Li C., Liu J., Xie Z. (2020). PD-L1 expression with respect to driver mutations in non-small cell lung cancer in an Asian population: a large study of 1370 cases in China. *Therapeutic Advances in Medical Oncology*.

[12] Lamberti G., Spurr L. F., Li Y. (2020). Clinicopathological and genomic correlates of programmed cell death ligand 1 (PD-L1) expression in nonsquamous non-small-cell lung cancer. *Annals of Oncology*.

[13] Reck M., Rodriguez-Abreu D., Robinson A. (2019). Updated analysis of KEYNOTE-024: pembrolizumab versus platinum-based chemotherapy for advanced non-small-cell lung cancer with PD-L1 tumor proportion score of 50% or greater. *Journal of Clinical Oncology*.

[14] Lopes G., Wu Y.-L., Kudaba I. (2018). Pembrolizumab (pembro) versus platinum-based chemotherapy (chemo) as first-line therapy for advanced/metastatic NSCLC with a PD-L1 tumor proportion score (TPS) ≥ 1%: open-label, phase 3 KEYNOTE-042 study. *Journal of Clinical Oncology*.

[15] Horn L., Spigel D. R., Vokes E. E. (2017). Nivolumab versus docetaxel in previously treated patients with advanced non–small-cell lung cancer: two-year outcomes from two randomized, open-label, phase III trials (CheckMate 017 and CheckMate 057). *Journal of Clinical Oncology*.

[16] Yang Z., Yang N., Ou Q. (2018). Investigating novel resistance mechanisms to third-generation EGFR tyrosine kinase inhibitor osimertinib in non-small cell lung cancer patients. *Clinical Cancer Research*.

[17] Jin Y., Bao H., Le X. (2020). Distinct co-acquired alterations and genomic evolution during TKI treatment in non-small-cell lung cancer patients with or without acquired T790M mutation. *Oncogene*.

[18] Bolger A. M., Lohse M., Usadel B. (2014). Trimmomatic: a flexible trimmer for Illumina sequence data. *Bioinformatics*.

[19] Li H., Durbin R. (2009). Fast and accurate short read alignment with burrows-wheeler transform. *Bioinformatics*.

[20] Van der Auwera G. A., Carneiro M. O., Hartl C. (2013). From FastQ data to high-confidence variant calls: the genome analysis toolkit best practices pipeline. *Current Protocols in Bioinformatics*.

[21] Cibulskis K., Lawrence M. S., Carter S. L. (2013). Sensitive detection of somatic point mutations in impure and heterogeneous cancer samples. *Nature Biotechnology*.

[22] Song Z., Cai Z., Yan J., Shao Y. W., Zhang Y. (2019). Liquid biopsies using pleural effusion-derived exosomal DNA in advanced lung adenocarcinoma. *Translational Lung Cancer Research*.

[23] Lan X., Bao H., Ge X. (2020). Genomic landscape of metastatic papillary thyroid carcinoma and novel biomarkers for predicting distant metastasis. *Cancer science*.

[24] Wu Y.-L., Zhang L., Fan Y. (2021). Randomized clinical trial of pembrolizumab vs chemotherapy for previously untreated Chinese patients with PD-L1-positive locally advanced or metastatic non–small-cell lung cancer: KEYNOTE-042 China Study. *International Journal of Cancer*.

[25] Rosell R., Gonzalez-Cao M. (2021). Cemiplimab monotherapy in advanced non-squamous and squamous non-small cell lung cancer. *The Lancet*.

[26] Büttner R., Gosney J. R., Skov B. G. (2017). Programmed death-ligand 1 immunohistochemistry testing: a review of analytical assays and clinical implementation in non-small-cell lung cancer. *Journal of Clinical Oncology*.

[27] Yun C. H., Boggon T. J., Li Y. (2007). Structures of lung cancer-derived EGFR mutants and inhibitor complexes: mechanism of activation and insights into differential inhibitor sensitivity. *Cancer Cell*.

[28] Wee P., Wang Z. (2017). Epidermal growth factor receptor cell proliferation signaling pathways. *Cancers*.

[29] Mehnert J. M., Panda A., Zhong H. (2016). Immune activation and response to pembrolizumab in POLE-mutant endometrial cancer. *The Journal of Clinical Investigation*.

[30] Hussein Y. R., Weigelt B., Levine D. A. (2015). Clinicopathological analysis of endometrial carcinomas harboring somatic POLE exonuclease domain mutations. *Modern Pathology*.

[31] Latchman Y., Wood C. R., Chernova T. (2001). PD-L2 is a second ligand for PD-1 and inhibits T cell activation. *Nature Immunology*.

[32] Hsieh C. C., Hsu H. S., Li A. F., Chen Y. J. (2018). Clinical relevance of PD-L1 and PD-L2 overexpression in patients with esophageal squamous cell carcinoma. *Journal of thoracic disease*.

[33] Rizvi H., Sanchez-Vega F., La K. (2018). Molecular determinants of response to anti–programmed cell death (PD)-1 and anti–programmed death-ligand 1 (PD-L1) blockade in patients with non–small-cell lung cancer profiled with targeted next-generation sequencing. *Journal of Clinical Oncology*.

[34] Chen Y., Liu Q., Chen Z. (2019). PD-L1 expression and tumor mutational burden status for prediction of response to chemotherapy and targeted therapy in non-small cell lung cancer. *Journal of Experimental & Clinical Cancer Research*.

[35] Schoenfeld A. J., Rizvi H., Bandlamudi C. (2020). Clinical and molecular correlates of PD-L1 expression in patients with lung adenocarcinomas^✰^. *Annals of Oncology*.

[36] Hong L., Dibaj S., Negrao M. V. (2019). Spatial and temporal heterogeneity of PD-L1 and its impact on benefit from immune checkpoint blockade in non-small cell lung cancer (NSCLC). *Journal of Clinical Oncology*.

[37] Chakravarty D., Gao J., Phillips S. (2017). Onco KB: a precision oncology knowledge base. *JCO Precision Oncology*.

[38] Champiat S., Dercle L., Ammari S. (2017). Hyperprogressive disease is a new pattern of progression in cancer patients treated by anti-PD-1/PD-L1. *Clinical Cancer Research*.

[39] Dong Z. Y., Zhong W. Z., Zhang X. C. (2017). Potential predictive value of TP53 and KRAS mutation status for response to PD-1 blockade immunotherapy in lung adenocarcinoma. *Clinical Cancer Research*.

[40] Assoun S., Theou-Anton N., Nguenang M. (2019). Association of TP53 mutations with response and longer survival under immune checkpoint inhibitors in advanced non-small-cell lung cancer. *Lung cancer*.

[41] Xu H., Lin G., Huang C. (2017). Assessment of concordance between 22C3 and SP142 immunohistochemistry assays regarding PD-L1 expression in non-small cell lung cancer. *Scientific Reports*.

[42] Parra E. R., Villalobos P., Mino B., Rodriguez-Canales J. (2018). Comparison of different antibody clones for immunohistochemistry detection of programmed cell death ligand 1 (PD-L1) on non-small cell lung carcinoma. *Applied immunohistochemistry & molecular morphology: AIMM.*.

[43] Azuma K., Ota K., Kawahara A. (2014). Association of PD-L1 overexpression with activating EGFR mutations in surgically resected nonsmall-cell lung cancer. *Annals of Oncology*.

[44] Schoenfeld A. J., Rizvi H., Bandlamudi C. (2020). Clinical and molecular correlates of PD-L1 expression in patients with lung adenocarcinomas. *Annals of oncology: official journal of the European Society for Medical Oncology.*.

[45] Zhang K., Wang G., Guo C. (2019). Mutational landscapes and PD-L1 expression in non-small cell lung cancer. *Journal of Clinical Oncology*.

[46] Xu X., Yang Y., Liu X. (2020). NFE2L2/KEAP1 mutations correlate with higher tumor mutational burden value/PD-L1 expression and potentiate improved clinical outcome with immunotherapy. *The Oncologist*.

[47] Sabari J., Leonardi G., Shu C. (2018). PD-L1 expression, tumor mutational burden, and response to immunotherapy in patients with MET exon 14 altered lung cancers. *Annals of Oncology*.

[48] Pawelczyk K., Piotrowska A., Ciesielska U. (2019). Role of PD-L1 expression in non-small cell lung cancer and their prognostic significance according to clinicopathological factors and diagnostic markers. *International Journal of Molecular Sciences*.

[49] Calles A., Liao X., Sholl L. M. (2015). Expression of PD-1 and its ligands, PD-L1 and PD-L2, in smokers and never smokers with KRAS-mutant lung cancer. *Journal of Thoracic Oncology*.

[50] Song P., Zhang J., Shang C., Zhang L. (2019). Real-world evidence and clinical observations of the treatment of advanced non-small cell lung cancer with PD-1/PD-L1 inhibitors. *Scientific Reports*.

[51] Zauderer M. G. (2019). Practical application of real-world evidence in developing cancer therapies. *JCO Clinical Cancer Informatics*.

[52] Rizvi H., Sanchez-Vega F., La K. (2018). Molecular determinants of response to anti-programmed cell death (PD)-1 and anti-programmed death-ligand 1 (PD-L1) blockade in patients with non-small-cell lung cancer profiled with targeted next-generation sequencing. *Journal of clinical oncology*.

