# Supplementary material for: Correlation of PD-L1 Expression with Clinicopathological and Genomic Features in Chinese Non-Small-Cell Lung Cancer
Source: J Oncol. 2022 Apr 11;2022:1763778. doi: 10.1155/2022/1763778 (PMC9015849; doi:10.1155/2022/1763778)
Supplement: Supplementary Materials — Supplementary Figure S1: representative images of immunohistochemical staining for PD-1 with 22C3 and 28-8 from ADC or SCC patients. (A-C) PD-L1 expression (clone 22C3) in ADC: (A) TPS < 1%, (B) TPS 1-49%, and (C) TPS ≥ 50%. (D-F) PD-L1 expression (clone 22C3) in SCC: (D) TPS < 1%, (E) TPS 1-49%, and (F) TPS ≥ 50%. (G-I) PD-L1 expression (clone 28-8) in ADC: (G) TPS < 1%, (H) TPS 1-9%, and (I) TPS ≥ 10%. (J-L) PD-L1 expression (clone 28-8) in SCC: (J) TPS < 1%, (K) TPS 1-9%, and (L) TPS ≥ 10%. PD-L1: programmed cell death-ligand 1; ADC: adenocarcinoma; SCC: squamous cell carcinoma; TPS: tumor proportion score. Scale bar: 100 μm. Supplementary Figure S2: scatter plot of PD-L1 expression (clone 22C3) with TMB in ADC. TMB does not correlate with PD-L1 expression (n = 464, Pearson's correlation = 0.060). Triangle represents specimen of each patient. Supplementary Table S1: gene list of 139 panel and 425 panel. Supplementary Table S2: genetic mutations and PD-L1 expression (clone 22C3) in ADC (n = 552) and SCC (n = 119) patient samples. Supplementary Table S3: gene copy number variations and PD-L1 expression (clone 22C3) in ADC (n = 464) and SCC (n = 119) patient samples. Supplementary Table S4: arm-level copy number variations and PD-L1 expression (clone 22C3) in ADC (n = 464) and SCC (n = 119) patient samples. Supplementary Table S5: genomic alterations and PD-L1 expression (clone 28-8) in ADC (n = 93) patient samples. [file 1763778.f1.zip › 1763778.f1/Supplementary table_PD-L1 and genomic alterations.docx]

**Supplementary Table S1. Gene list of 139 panel and 425 panel.**

| **Panel** | **Gene** |
| --- | --- |
| 139 | *AKT1, AKT2, AKT3, ALK, APC, AR, ARAF, ARID1A, ARID2, ASXL1, ATM, ATR, ATRX, AXL, BCL2L11 (BIM), BRAF, BRCA1, BRIP1, BTK, CD274 (PD-L1), CD74, CDA, CDH1, CDK4, CDK6, CDK8, CDKN1B, CDKN2A, CDKN2B, CHEK2, CREBBP, CTNNB1, CYLD, CYP2B6, CYP2C19, CYP2D6, CYP3A4, CYP3A5, DDR2, DHFR, DNMT3A, DPYD, EGFR, ERBB2 (HER2), ERBB3, ERBB4, ERCC1, ERCC2, ERCC4, FAT1, FBXW7, FGFR1, FGFR3, FLT4, FRG1, GATA4, GNAS, GRIN2A, GSTM1, GSTP1, GSTT1, HDAC9, HGF, HRAS, IDH1, IDH2, JAK1, JAK2, KDR (VEGFR2), KEAP1, KIT, KMT2A (MLL), KMT2C, KMT2D, KRAS, LRP1B, LZTR1, MAP2K1 (MEK1), MAP2K2 (MEK2), MED12, MET, MLH1, MTHFR, MTOR, MYC, NBN, NF1, NF2, NFE2L2, NOTCH1, NQO1, NRAS, NTRK1, NTRK3, PBRM1, PDCD1 (PD1) , PDCD1LG2 (PD-L2), PDGFRA, PDGFRB, PIK3CA, PIK3CD, PIK3R1, PTEN, PTPN11, QKI, RAF1, RB1, RECQL4, RELN, RET, RHOA, RICTOR, ROS1, SBDS, SDC4, SETD2, SF3B1, SLC34A2, SMAD2, SMAD3, SMAD4, SMARCA4, SMARCB1, SOX2, STAG2, STAT3, STK11, TET2, TGFBR2, TP53, TPMT, TSC1, TSC2, TYMS, U2AF1, UGT1A1, VEGFA, WRN, XRCC1* |
| 425 | *ABCB1 (MDR1), ABCB4, ABCC2 (MRP2), ADH1A, ADH1B, ADH1C, AIP, AKT1, AKT2, AKT3, ALDH2, ALK, AMER1, APC, AR, ARAF, ARID1A, ARID1B, ARID2, ARID5B, ASCL4, ASXL1, ATF1, ATIC, ATM, ATR, ATRX, AURKA, AURKB, AXIN2, AXL, B2M, BAD, BAI3, BAK1, BAP1, BARD1, BAX, BCL2, BCL2L11 (BIM), BCR, BIRC3, BLM, BMPR1A, BRAF, BRCA1, BRCA2, BRD4, BRIP1, BTG2, BTK, BUB1B, CLLORF30, CASP8, CBL, CBLB, CCND1, CCNE1, CD274 (PD-L1), CD74, CDA, CDC73, CDH1, CDK10, CDK12, CDK4, CDK6, CDK8, CDKN1A, CDKN1B, CDKN1C, CDKN2A, CDKN2B, CDKN2C, CEBPA, CEP57, CHD4, CHEK1, CHEK2, CREBBP, CRKL, CSF1R, CTCF, CTLA4, CTNNB1, CUL3, CUX1, CXCR4, CYLD, CYP19A1, CYP2A13, CYP2A6, CYP2A7, CYP2B6*6, CYP2C19*2, CYP2C9*3, CYP2D6, CYP3A4*4, CYP3A5, DAXX, DDR2, DENND1A, DHFR, DICER1, DLL3, DNMT3A, DPYD, DUSP2, EGFR, EML4, EP300, EPAS1, EPCAM, EPHA2, EPHA3, EPHA5, EPHB2, ERBB2 (HER2), ERBB2IP, ERBB3, ERBB4, ERCC1, ERCC2, ERCC3, ERCC4, ERCC5, ESR1, ETV1, ETV4, ETV6, EWSR1, EXT1, EXT2, EZH2, FANCA, FANCC, FANCD2, FANCE, FANCF, FANCG, FANCI, FANCL, FANCM, FAT1, FBXW7, FGF19, FGFR1, FGFR2, FGFR3, FGFR4, FH, FLCN, FLT1 (VEGFR1), FLT3, FLT4, FOXA1, FOXP1, FRG1, GATA1, GATA2, GATA3, GATA4, GATA6, GNA11, GNAQ, GNAS, GRIN2A, GRM3, GRM8, GSTM1, GSTM4, GSTM5, GSTP1, GSTT1, HDAC2, HDAC9, HGF, HLA-A, HNF1A, HNF1B, HRAS, HSD3B1, IDH1, IDH2, IFNG, IFNGR1, IGF1R, IGF2, IKBKE, IKZF1, IL7R, INPP4B, IRF2, JAK1, JAK2, JAK3, JARID2, JUN, KDM5A, KDM6A, KDR (VEGFR2), KEAP1, KIF1B, KIF5B, KIT, KITLG, KLLN, KMT2A (MLL), KMT2B, KMT2C, KMT2D, KRAS, LHCGR, LMO1, LRP1B, LYN, LZTR1, MAP2K1 (MEK1), MAP2K2 (MEK2), MAP2K4, MAP3K1, MAP3K4, MAP4K3, MAX, MCL1, MDM2, MDM4, MECOM, MED12, MEF2B, MEN1, MET, MGMT, MITF, MLH1, MLH3, MLLT1, MLLT3, MLLT4, MPL, MRE11A, MSH2, MSH6, MTHFR, MTOR, MUTYH, MYC, MYC, MYCN, MYD88, MYH9, NAT1, NBN NCOR1, NF1, NF2, NFE2L2, NFKBIA, NKX2-1, NKX2-4, NOTCH1, NOTCH2, NOTCH3, NPM1, NQO1, NRAS, NRG1, NSD1, NTRK1, NTRK2, NTRK3, PAK3, PALB2, PALLD, PARK2, PARP1, PARP2, PAX5, PBRM1, PDCD1 (PD1), PDCD1LG2 (PD-L2), PDE11A, PDGFRA, PDGFRB, PDK1, PGR, PHOX2B, PIK3C3, PIK3CA, PIK3R1, PIK3R2, PKHD1, PLAG1, PLK1, PMS1, PMS2, POLD1, POLD3, POLE, POLH, POT1, PPARD, PPP2R1A, PRDM1, PRF1, PRKACA, PRKACG, PRKAR1A, PRKCI, PRKDC, PRSS1, PRSS3, PTCH1, PTEN, PTK2, PTPN11, PTPN13, PTPRD, QKI, RAC1, RAC3, RAD50, RAD51, RAD51B, RAD51C, RAD51D, RAD54L, RAF1, RARA, RARG, RASGEF1A, RB1, RECQL4, RELN, RET, RHOA, RICTOR, RNF43, ROS1, RPTOR, RRM1, RUNX1, RUNX1T1, SBDS, SDC4, SDHA, SDHB, SDHC, SDHD, SEPT9, SETBP1, SETD2, SF3B1, SGK1, SLC34A2, SLC3A2, SLC7A8, SMAD2, SMAD3, SMAD4, SMAD7, SMARCA4, SMARCB1, SMO, SOS1, SOX1, SOX14, SOX2, SOX21, SPOP, SPRY4, SRC, SRY, STAG2, STAT3, STK11, STMN1, STT3A, SUFU, TAP1, TAP2, TEK, TEKT4, TERC, TERT, TET2, TGFBR2, THADA, TMEM127, TMPRSS2, TNFAIP3, TNFRSF11A, TNFRSF14, TNFRSF19, TNFSF11, TOP1, OP2A, TP53, TP63, TPMT, TSC1, TSC2, TSHR, TTF1, TUBB3, TUBB4A, TUBB4B, TUBB6, TYMS, U2AF1, UGT1A1, VAMP2, VEGFA, VHL, WAS, WISP3, WRN, WT1, XPA, XPC, XRCC1, YAP1, ZNF2, ZNF217, ZNF703* |

**Supplementary Table S2.** Genetic mutations and PD-L1 expression (clone 22C3) in ADC (n = 552) and SCC (n = 119) patient samples.

| **Gene** | **PD-L1 (clone 22C3) TPS** | | | | | | | | | | | | |
| --- | --- | --- | --- | --- | --- | --- | --- | --- | --- | --- | --- | --- | --- |
|  | **Group1** | | | | **Group2** | | | | **Group3** | | | | |
|  | **< 1%** | **≥ 1%** | ***p-value*** | **adj.*p*** | **< 50%** | **≥ 50%** | ***p-value*** | **adj.*p*** | **< 1%** | **1-49%** | **≥ 50%** | ***p-value*** | **adj.*p*** |
| **ADC: mutations** (n = 464) | | | | | | | | | | | | | |
| ***TP53*** (n=331) | 152 | 179 | **< 0.001** | **0.007** | 271 | 60 | **0.015** | **0.089** | 152 | 119 | 60 | **< 0.001** | **0.004** |
| ***EGFR*** (n=291) | 165 | 126 | **0.027** | **0.12** | 264 | 27 | **< 0.001** | **0.001** | 165 | 99 | 27 | **< 0.001** | **0.004** |
| ***KRAS*** (n=94) | 37 | 57 | **0.007** | **0.06** | 70 | 24 | **0.004** | **0.035** | 37 | 33 | 24 | **< 0.001** | **0.004** |
| ***RET*** (n=10) | 9 | 1 | **0.022** | **0.12** | 10 | 0 | 0.372 | 0.694 | 9 | 1 | 0 | **0.021** | **0.094** |
| ***POLE*** (n=12) | 10 | 2 | **0.039** | **0.139** | 11 | 1 | 1 | 1 | 10 | 1 | 1 | 0.07 | **0.209** |
| **ADC: oncogenic mutations** (n = 464) | | | | | | | | | | | | | |
| ***TP53*** (n=127) | 63 | 64 | 0.544 | 0.855 | 107 | 20 | 0.779 | 0.779 | 63 | 44 | 20 | 0.564 | 0.755 |
| ***EGFR*** (n=264) | 152 | 112 | **0.017** | **0.063** | 240 | 24 | **< 0.001** | **0.002** | 152 | 88 | 24 | **< 0.001** | **0.004** |
| L858R (n=123) | 63 | 60 | 0.752 | 0.919 | 113 | 10 | **0.004** | **0.015** | 63 | 50 | 10 | 0.094 | **0.173** |
| 19Del (n=97) | 61 | 36 | **0.021** | **0.063** | 88 | 9 | **0.021** | **0.047** | 61 | 27 | 9 | **0.007** | **0.018** |
| Other (n=44) | 28 | 16 | 0.079 | **0.127** | 39 | 5 | 0.298 | 0.365 | 28 | 11 | 5 | 0.07 | **0.154** |
| ***KRAS*** (n=90) | 36 | 54 | **0.015** | **0.063** | 66 | 24 | **0.002** | **0.01** | 36 | 30 | 24 | **< 0.001** | **0.004** |
| G12C (n=73) | 29 | 44 | **0.023** | **0.063** | 54 | 19 | **0.007** | **0.018** | 29 | 25 | 19 | **0.002** | **0.007** |
| ***KIF5B-RET*** (n=6) | 0 | 6 | **0.012** | **0.047** | 3 | 3 | **0.047** | **0.187** | 0 | 3 | 3 | **0.003** | **0.228** |
| **SCC: mutations** (n = 119) | | | | | | | | | | | | | |
| ***NFE2L2*** (n=20) | 5 | 15 | **0.032** | 0.259 | 12 | 8 | **0.007** | **0.105** | 5 | 7 | 8 | **0.003** | **0.046** |
| ***APC*** (n=15) | 12 | 3 | **0.012** | **0.192** | 15 | 0 | 0.075 | **0.238** | 12 | 3 | 0 | **0.007** | **0.052** |
| ***POLE*** (n=9) | 2 | 7 | 0.171 | 0.617 | 5 | 4 | **0.044** | **0.232** | 2 | 3 | 4 | **0.027** | **0.142** |
| ***ALK*** (n=9) | 3 | 6 | 0.499 | 0.967 | 5 | 4 | **0.044** | **0.232** | 3 | 2 | 4 | 0.08 | **0.213** |
| **SCC: oncogenic mutations** (n = 119) | | | | | | | | | | | | | |
| ***TP53*** (n=45) | 27 | 18 | **0.049** | 0.294 | 35 | 10 | 0.24 | 0.395 | 27 | 8 | 10 | 0.443 | 0.899 |

Abbreviations: PD-L1, programmed cell death-ligand 1; TPS, tumor proportion score; ADC, adenocarcinoma; SCC, squamous cell carcinoma; adj.*p*, FDR adjust *p*-value.

**Supplementary Table S3.** Gene copy number variations and PD-L1 expression (clone 22C3) in ADC (n = 464) and SCC (n = 119) patient samples.

| **Gene** | **PD-L1 (clone 22C3) TPS** | | | | | | | | | | | | |
| --- | --- | --- | --- | --- | --- | --- | --- | --- | --- | --- | --- | --- | --- |
|  | **Group1** | | | | **Group2** | | | | **Group3** | | | | |
|  | **< 1%** | **≥ 1%** | ***p-value*** | **adj.*p*** | **< 50%** | **≥ 50%** | ***p-value*** | **adj.*p*** | **< 1%** | **1-49%** | **≥ 50%** | ***p-value*** | **adj.*p*** |
| **ADC** (n = 464) | | | | | | | | | | | | | |
| ***MDM2* Gain** (n=28) | 19 | 9 | 0.119 | 0.501 | 28 | 0 | **0.0220** | **0.234** | 19 | 9 | 0 | **0.027** | 0.293 |
| ***PD-L1* Gain** (n=10) | 0 | 10 | **< 0.001** | **0.021** | 4 | 6 | **< 0.001** | **0.026** | 0 | 4 | 6 | **< 0.001** | **< 0.001** |
| ***PD-L2* Gain** (n=7) | 0 | 7 | **< 0.001** | **0.016** | 3 | 4 | **0.009** | **0.142** | 0 | 3 | 4 | **< 0.001** | 0.662 |
| **SCC** (n = 119) | | | | | | | | | | | | | |
| ***PTPRD* Loss** (n=13) | 10 | 3 | **0.037** | 0.404 | 13 | 0 | 0.121 | 0.652 | 10 | 3 | 0 | **0.015** | **0.234** |
| ***MDM2* Gain** (n=9) | 7 | 2 | 0.082 | 0.436 | 9 | 0 | 0.358 | 0.711 | 7 | 2 | 0 | **0.044** | **0.234** |
| ***EGFR* Gain** (n=7) | 6 | 1 | 0.051 | 0.404 | 7 | 0 | 0.351 | 0.711 | 6 | 1 | 0 | **0.040** | **0.234** |

Abbreviations: PD-L1, programmed cell death-ligand 1; ADC, adenocarcinoma; SCC, squamous cell carcinoma; adj.p, FDR adjust p-value.

**Supplementary Table S4.** Arm level copy number variations and PD-L1 expression (clone 22C3) in ADC (n = 464) and SCC (n = 119) patient samples.

| Arm | PD-L1 (clone 22C3) TPS | | | | | | | | | | | |  | |
| --- | --- | --- | --- | --- | --- | --- | --- | --- | --- | --- | --- | --- | --- | --- |
|  | **Group1** | | | | **Group2** | | | | **Group3** | | | | | |
|  | **< 1%** | **≥ 1%** | ***p*-value** | **adj.*p*** | **< 50%** | **≥ 50%** | ***p*-value** | **adj.*p*** | **< 1%** | **1–49%** | **≥ 50%** | ***p*-value** | **adj.*p*** |  |
| ADC (n = 464) | | | | | | | | | | | | | |  |
| chr.1q Amp (n=107) | 62 | 45 | 0.270 | 0.809 | 103 | 4 | **< 0.001** | **0.014** | 62 | 41 | 4 | **0.012** | 0.398 |  |
| chr.12p Amp (n=51) | 22 | 29 | 0.180 | 0.752 | 39 | 12 | 0.052 | 0.878 | 15 | 7 | 5 | **0.044** | 0.560 |  |
| chr.13q Amp (n=15) | 12 | 3 | **0.036** | 0.752 | 14 | 1 | 0.706 | 1.000 | 12 | 2 | 1 | 0.059 | 0.560 |  |
| chr.20q Amp (n=162) | 85 | 77 | 0.923 | 1.000 | 147 | 15 | **0.036** | 0.807 | 85 | 62 | 15 | 0.096 | 0.783 |  |
| chr.1p Del (n=16) | 12 | 4 | 0.080 | 0.752 | 16 | 0 | 0.144 | 0.893 | 12 | 4 | 0 | **0.041** | 0.560 |  |
| chr.5q Del (n=30) | 23 | 7 | **0.008** | 0.524 | 29 | 1 | 0.102 | 0.878 | 23 | 6 | 1 | **0.007** | 0.398 |  |
| chr.12p Del (n=30) | 21 | 9 | 0.059 | 0.752 | 29 | 1 | 0.102 | 0.878 | 21 | 8 | 1 | **0.029** | 0.560 |  |
| SCC (n = 119) | | | | | | | | | | | | | |  |
| chr.9p Amp (n=6) | 0 | 6 | **0.029** | 0.615 | 3 | 3 | 0.067 | 0.802 | 0 | 3 | 3 | **0.008** | **0.211** |  |
| chr.14q Amp (n=19) | 13 | 6 | **0.048** | 0.615 | 18 | 1 | 0.190 | 0.942 | 13 | 5 | 1 | **0.032** | 0.294 |  |
| chr.20q Amp (n=39) | 23 | 16 | 0.081 | 0.615 | 36 | 3 | 0.071 | 0.802 | 23 | 13 | 3 | **0.026** | 0.287 |  |
| chr.13q Del (n=37) | 12 | 25 | **0.047** | 0.615 | 27 | 10 | 0.117 | 0.802 | 12 | 15 | 10 | **0.019** | 0.287 |  |
| chr.19p Del (n=14) | 10 | 4 | 0.085 | 0.615 | 14 | 0 | 0.127 | 0.802 | 10 | 4 | 0 | **0.025** | 0.287 |  |

Abbreviations: PD-L1, programmed cell death-ligand 1; TPS, tumor proportion score; ADC, adenocarcinoma; SCC, squamous cell carcinoma; adj.*p*, FDR adjust *p*-value.

**Supplementary Table S5.** Genomic alterations and PD-L1 expression (clone 28-8) in ADC (n = 93) patient samples.

| Arm | PD-L1 (clone 28-8) TPS | | | | | | | | | | | |  | |
| --- | --- | --- | --- | --- | --- | --- | --- | --- | --- | --- | --- | --- | --- | --- |
|  | **Group1** | | | | **Group2** | | | | **Group3** | | | | | |
|  | **< 1%** | **≥ 1%** | ***p*-value** | **adj.*p*** | **< 10%** | **≥ 10%** | ***p*-value** | **adj.*p*** | **< 1%** | **1–9%** | **≥ 10%** | ***p*-value** | **adj.*p*** |  |
| ADC (n = 93) | | | | | | | | | | | | | |  |
| *NKX2-1* Gain (n=107) | 62 | 45 | 0.270 | 0.809 | 103 | 4 | **< 0.001** | **0.014** | 62 | 41 | 4 | **0.012** | 0.398 |  |
| chr.9q Del (n=11) | 8 | 3 | **0.046** | 0.955 | 8 | 4 | 0.756 | 0.965 | 8 | 1 | 2 | **0.038** | 0.772 |  |
| chr.19q Del (n=7) | 2 | 5 | 1.000 | 1.000 | 7 | 0 | **0.039** | 0.561 | 2 | 5 | 0 | 0.406 | 0.913 |  |
| chr.19p Del (n=18) | 8 | 10 | 0.695 | 0.955 | 15 | 3 | **0.031** | 0.561 | 8 | 7 | 3 | 0.167 | 0.780 |  |
| chr.1p Del (n=16) | 12 | 4 | 0.080 | 0.752 | 16 | 0 | 0.144 | 0.893 | 12 | 4 | 0 | **0.041** | 0.560 |  |
| chr.5q Del (n=30) | 23 | 7 | **0.008** | 0.524 | 29 | 1 | 0.102 | 0.878 | 23 | 6 | 1 | **0.007** | 0.398 |  |
| chr.12p Del (n=30) | 21 | 9 | 0.059 | 0.752 | 29 | 1 | 0.102 | 0.878 | 21 | 8 | 1 | **0.029** | 0.560 |  |

Abbreviations: PD-L1, programmed cell death-ligand 1; TPS, tumor proportion score; ADC, adenocarcinoma; adj.*p*, FDR adjust *p*-value.
