# Supplementary material for: Correlation of PD-L1 Expression with Clinicopathological and Genomic Features in Chinese Non-Small-Cell Lung Cancer
Source: J Oncol. 2022 Apr 11;2022:1763778. doi: 10.1155/2022/1763778 (PMC9015849; doi:10.1155/2022/1763778)

Supplementary Figure S1

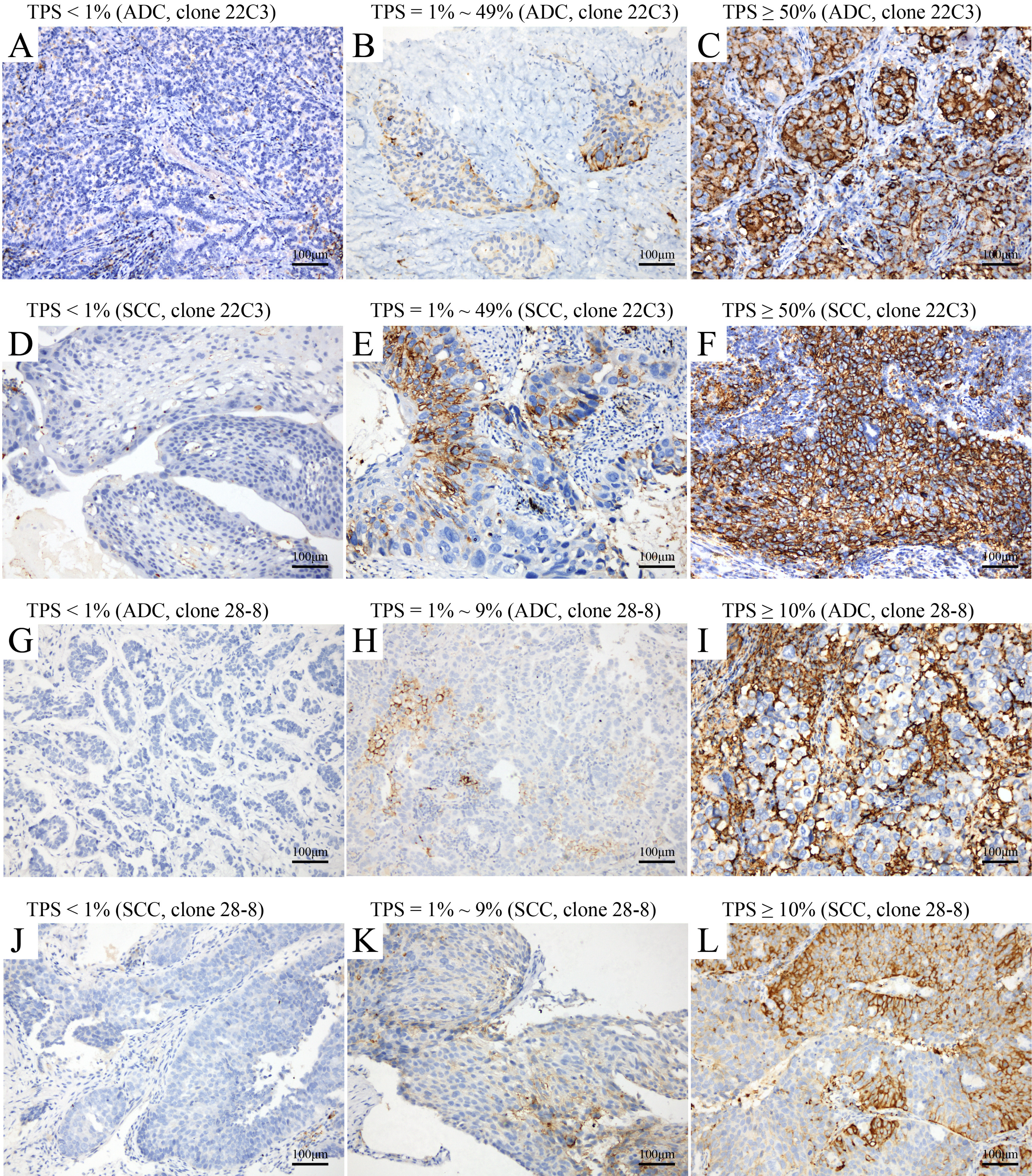

Supplement: Supplementary Materials — Supplementary Figure S1: representative images of immunohistochemical staining for PD-1 with 22C3 and 28-8 from ADC or SCC patients. (A-C) PD-L1 expression (clone 22C3) in ADC: (A) TPS < 1%, (B) TPS 1-49%, and (C) TPS ≥ 50%. (D-F) PD-L1 expression (clone 22C3) in SCC: (D) TPS < 1%, (E) TPS 1-49%, and (F) TPS ≥ 50%. (G-I) PD-L1 expression (clone 28-8) in ADC: (G) TPS < 1%, (H) TPS 1-9%, and (I) TPS ≥ 10%. (J-L) PD-L1 expression (clone 28-8) in SCC: (J) TPS < 1%, (K) TPS 1-9%, and (L) TPS ≥ 10%. PD-L1: programmed cell death-ligand 1; ADC: adenocarcinoma; SCC: squamous cell carcinoma; TPS: tumor proportion score. Scale bar: 100 μm. Supplementary Figure S2: scatter plot of PD-L1 expression (clone 22C3) with TMB in ADC. TMB does not correlate with PD-L1 expression (n = 464, Pearson's correlation = 0.060). Triangle represents specimen of each patient. Supplementary Table S1: gene list of 139 panel and 425 panel. Supplementary Table S2: genetic mutations and PD-L1 expression (clone 22C3) in ADC (n = 552) and SCC (n = 119) patient samples. Supplementary Table S3: gene copy number variations and PD-L1 expression (clone 22C3) in ADC (n = 464) and SCC (n = 119) patient samples. Supplementary Table S4: arm-level copy number variations and PD-L1 expression (clone 22C3) in ADC (n = 464) and SCC (n = 119) patient samples. Supplementary Table S5: genomic alterations and PD-L1 expression (clone 28-8) in ADC (n = 93) patient samples. [file 1763778.f1.zip › 1763778.f1/Supplemnetary Figure S1 PD-L1 testing(22C3+28-8).pdf]
